# Comparing the effects of fluoxetine and imipramine on total cholesterol, triglyceride, and weight in patients with major depression

**DOI:** 10.1186/2008-2231-21-4

**Published:** 2013-01-05

**Authors:** Esmaeil Shahsavand Ananloo, Padideh Ghaeli, Mohammad-Zaman Kamkar, Majid Sadeghi

**Affiliations:** 1 Department of Genomic Psychiatry (DGP), Roozbeh Hospital, Tehran University of Medical Sciences (TUMS), Tehran, IRAN; 2 Roozbeh Hospital, Tehran University of Medical Sciences (TUMS), Tehran, IRAN; 3 Fifth of Azar Hospital, Golestan University of Medical Sciences, Gorgan, IRAN; 4 Psychiatry and Psychology Research Center, Roozbeh Hospital, Tehran University of Medical Sciences (TUMS), Tehran, IRAN

**Keywords:** Tricyclic antidepressants, Serotonin reuptake inhibitors, Cholesterol, Triglyceride, Body weight

## Abstract

**Background:**

There are some reports on the effects of antidepressants on metabolic syndrome. However, our search in the previously published literature showed a lack of information on the comparison of the effects of different classes of antidepressants on lipid profile. Therefore, this study was aimed to compare the effects of fluoxetine and imipramine on serum total cholesterol (TC) and triglyceride (TG) as well as body weight (BW) in patients with major depressive disorder.

**Methods:**

Fifty one patients, 18 to 70 years of age, with major depressive disorder complied with the criteria of this preliminary, open-label clinical trial. Subjects received either imipramine (75–200 mg/day) or fluoxetine (20–40 mg/day) for 8 weeks. Total cholesterol and TG levels, as well as BW were compared at baseline with those at weeks 4 and 8. Data was analyzed by SPSS software version 16.0.

**Results:**

In the fluoxetine group, TC levels decreased from 165.71 mg/dL to 156.71 mg/dL at week 4 (P = 0.07), and to 143.94 mg/dL at week 8 (P = 0.16); TG levels decreased from 129.35 mg/dL to 115.88 mg/dL at week 4 (P <0.001), and to 110.41 mg/dL at week 8 (P = 0.56). In the imipramine group, TC levels increased from 169.10 mg/dL to 178.69 mg/dL at week 4 (P = 0.07), and to 208.69 mg/dL at week 8 (P < 0.001) while TG levels increased from 111.73 mg/dL to 128.83 mg/dL at week 4 (P = 0.005), and to 160.90 mg/dL at week 8 (P < 0.001). BW was significantly increased in the imipramine group at weeks 4 and 8. In the fluoxetine group, BW was non-significantly decreased from 75.69 ± 7.97 Kg (baseline) to 75.67 ± 8.01 Kg at week 4 (P = 0.88), and to 75.22 ± 8.67 Kg at week 8 (P = 0.20), while in the imipramine group, BW had significant increases from 72.53 ± 8.55 Kg (baseline) to 73.95 ± 8.61 mg/dL at week 4 (P < 0.001), and to 75.13 ± 8.34 mg/dL at week 8 (P < 0.001).

Repeated measures ANOVA showed significant effects on both TC and TG levels as well as on BW in all patients receiving imipramine. However, in patients on fluoxetine, repeated measures ANOVA showed significant effects of this medication only on TC levels in males.

**Conclusions:**

Monitoring TC and TG and BW is recommended before starting imipramine in depressed patients with increased risk for cardiovascular disease. Fluoxetine may be the preferred agent in those with high or borderline high lipid levels.

## Background

The lifetime prevalence of depressive disorders has been reported to be high. National Comorbidity Survey (NCS), conducted in 1990–1992, noted that the lifetime prevalence of major depressive disorder in the United States was 14.9% for lifetime and 8.6% for 12-month [1]. However, after the introduction of the DSM-IV criteria, the National Comorbidity Survey Replication (NCS-R) reported the prevalence of MDD as 16.2% for lifetime and 6.6% for 12-month by using World Health Organization’s Composite International Diagnostic Interview (CIDI) [2,3].

Fluoxetine, a specific serotonin reuptake inhibitor (SSRI) and imipramine, a tricyclic antidepressant (TCA) have been successfully used in the treatment of major depression for many years. These drugs differ in their pharmacology, adverse effects, and drug-drug interactions. Fluoxetine is well absorbed after oral administration and has a large volume of distribution. It is extensively metabolized in the liver, and is highly protein bound. Fluoxetine has an elimination half-life (t ½) of 24 to 96 hours; its metabolite t ½, norfluoxetine, ranges between 168 and 360 hours. Therefore, this drug should be used with caution in patients with decreased liver function and metabolic activity [4,5]. On the other hand, imipramine is highly metabolized in the liver with a t ½ of 6 to 28 hours; its major active metabolite is desipramine. This drug blocks histamine-1 and alpha-1 receptors and therefore may cause sedation, increase appetite, and orthostatic hypotension. It may also cause constipation, blurred vision, and urinary retention due to its anticholinergic effects [5].

Cipriani et al. analyzed data from 102 randomized clinical trials (RCTs) that compared SSRIs and TCAs, and noted no overall difference in efficacy between these two classes of antidepressants. However, SSRIs were shown to be better tolerated than TCAs according to the results of this analysis [6].

Since weight changes are among the criteria of depression and may result in related consequences such as alteration in lipid profile, this preliminary study was aimed to observe and compare the effects of fluoxetine and imipramine (representatives of the two classes of SSRIs and TCAs) on serum total cholesterol (TC) and triglyceride (TG), as well as body weight (BW) in depressed patients.

On the other hand, a number of factors such as medications, appetite, exercise, and medical illnesses may be of importance regarding weight changes associated with depression [7].

After searching Medline, and PsychLit from 1970 to 2012, we found that there are a few studies on the effects of fluoxetine or imipramine on serum TC, TG, and BW in animals and even fewer reports in depressed patients. For instance, one study in 1976 reported that prolonged intragastric administration of imipramine in rabbits resulted in a significant increase in serum cholesterol after 4, 8 and 12 weeks [8]. Additionally, association between TCAs and weight gain as well as association between SSRIs with abdominal obesity and hypercholesterolemia have been noted in the literature [9-12].

Activation of sterol regulatory element-binding protein (SREBP) may be important in understanding metabolic side effects of psychiatric drugs. Raeder et al. exposed cultured human glioma cells to some psychotropics. Some antidepressants including Imipramine and fluoxetine resulted in activation of SREBP system. Subsequently, it was noted that downstream genes that were involved in the biosynthesis of cholesterol and fatty acid were upregulated by studied antidepressants but to a different extent [13].

Based on the knowledge of the authors of the present study, no previous published study looked at the comparison of any SSRIs with TCAs regarding their effects on TG and TC levels and their relationships with BW in patients with major depressive disorder. Therefore, this preliminary study was designed to compare the effects of short-term use of fluoxetine and imipramine on TC and TG levels as well as BW in patients who suffered from major depressive disorder.

## Methods

Fifty eight patients, 18 to 70 years of age, with major depressive disorder (based on DSM-IV criteria) accepted to enter this preliminary, open-label, clinical trial. Subjects received either imipramine or fluoxetine for 8 weeks in private psychiatric offices in Tehran, Iran. Patients did not receive any antidepressant or any other medication(s) that was reported to affect lipid levels or BW (e.g., TCAs, antipsychotics, etc.) for 2 weeks before the initiation and during the study. Patients with TC levels above 250 mg/dL (according to the reference of the laboratory values), and TG levels above 200 mg/dL (according to the reference of the laboratory values), diabetes mellitus, a history of any major cardiovascular diseases, and pregnant women were excluded from this study. Additionally, none of the patients should have received electroconvulsive therapy within 6 months before the initiation of the antidepressants. Participants were asked to report any changes in their diet or appetite during the study. Patients were assigned to receive oral fluoxetine (20 to 40 mg/d) or imipramine (75 to 200 mg/d) for 8 weeks. Benzodiazepines were allowed when needed for anxiety, agitation, or sleep problems. Total cholesterol and TG levels as well as BW were measured at the initiation time as well as 4 and 8 weeks after starting antidepressants.

Fifty one out of the 58 patients, who signed informed consent forms, fulfilled the criteria of and participated in the study. The study was approved by the Ethics Committee of the Faculty of Pharmacy at Tehran University of Medical Sciences (TUMS) and was conducted in accordance with the Declaration of Helsinki 1975 as revised in 2000.

SPSS software version 16.0 was used to analyze the data. Independent-sample t-test was performed to compare the means of TC, TG, and BW measures between the 2 groups at baseline, weeks 4 and 8. Additionally, paired sample t-test was performed to compare the means of TC and TG levels, and also BW at weeks 0, 4, and 8 for each group.

Linear regression was used for each group to find whether or not the sex or age has any significant effects on TC or TG levels as well as BW. Repeated measures ANOVA was used to compare the results of TC and TG levels as well as BW between weeks 0, 4, and 8 in each groups. Results are presented as mean ± standard deviation (SD); standard error mean (SEM). Differences were considered significant when P < 0.05.

## Results

Fifty one patients complied with the study criteria and completed the study. Nineteen patients including 11 males and 8 females (mean age ± SD; SEM = 42.58 ± 15.19; 3.48 years) received fluoxetine while 32 patients including 13 males and 19 females (mean age ± SD; SEM = 41.97 ± 14.79; 2.61 years) received imipramine. The difference in age between the two groups was not significant (F = 0.017, P = 0.888). Baseline clinical characteristics of patients are shown in Table 1. As shown in this table, the differences between fluoxetine and imipramine groups at baseline for TC and TG levels as well as for BW were not statistically significant.

**Table 1:** Baseline clinical characteristics of patients

	**Imipramine**	**Fluoxetine**	**Statistics**
**Age (years); Mean ± SD**	41.97 ± 14.79	42.58 ± 15.19	F = 0.017, P = 0.888
**Age Range (years)**	18 – 70	18 - 68	
**TC (mg/dL)**	169.10 ± 36.20	165.71 ± 35.80	F = 0.088, P = 0.715
**TG (mg/dL)**	111.73 ± 36.52	129.35 ± 26.61	F = 0.088, P = 0.715
**BW (Kg)**	72.53 ± 8.55	75.69 ± 7.97	F = 0.059, P = 0.114

TC = Total Cholesterol; TG = Triglyceride; BW = Body Weight.

We did not find any reportable changes in patients’ appetite and food intake during the study; this finding was based on patients’ responses to a set of formatted questions about hydrocarbate, lipid and protein intake as well as appetite at every visit.

The results of repeated measure ANOVA and paired t-test for TC or TG level as well as for BW are shown in Table 2.

**Table 2:** Effects of Imipramine and Fluoxetine on serum TC and TG levels, and BW

**Groups**	**Paired t-test; Within groups changes between weeks 0 and 4 (p value)**	**Paired t-test; Within groups changes between weeks 4 and 8 (p value)**	**Repeated measures ANOVA; F (p value)**
**TC level (mg/dL)**			
Imipramine			
√ All subjects	9.59 (0.07**)	30.00 (<0.001*)	24.68 (<0.001*)
o Male	11.54 (0.21)	22.92 (<0.001*)	11.07 (<0.001*)
o Female	8.00 (0.22)	35.75 (0.003*)	13.88 (<0.001*)
Fluoxetine			
√ All subjects	9.00 (0.07**)	12.77 (0.16)	2.93 (0.07**)
o Male	6.80 (0.39)	19.10 (0.006*)	6.67 (0.027*)
o Female	12.14 (0.04*)	3.72 (0.86)	0.22 (0.810)
**TG level (mg/dL)**			
Imipramine			
√ All subjects	17.10 (0.005*)	32.07 (<0.001*)	18.88 (<0.001*)
o Male	11.75 (0.013*)	26.75 (<0.001*)	24.06 (<0.001*)
o Female	20.66 (0.036*)	35.62 (0.007*)	9.53 (0.001*)
Fluoxetine			
√ All subjects	13.47 (<0.001*)	5.47 (0.56)	2.59 (0.09)
o Male	14.27 (0.016*)	9.36 (0.30)	3.24 (0.06**)
o Female	12.00 (<0.001*)	1.67 (0.94)	0.26 (0.78)
**BW (Kg)**			
Imipramine			
√ All subjects	1.42 (<0.001*)	1.18 (<0.001*)	29.70 (<0.001*)
o Male	1.07 (0.009*)	1.00 (0.007*)	11.56 (<0.001*)
o Female	1.66 (<0.001*)	1.31 (0.008*)	18.54 (0.001*)
Fluoxetine			
√ All subjects	0.02 (0.88)	0.45 (0.20)	1.51 (0.24)
o Male	0.18 (0.44)	0.05 (0.85)	0.41 (0.54)
o Female	0.36 (0.25)	1.21 (0.14)	4.06 (0.05**)

* Statistically significant effect ** Trend towards significant effect.

As shown in Table 2, repeated measures ANOVA showed significant effects on both TC and TG levels as well as on BW in all patients receiving imipramine and also in males or females on the same antidepressant. However, in patients on fluoxetine, repeated measures ANOVA showed significant effects of this medication only on TC levels in males.

### TC levels analysis

Mean TC levels were significantly different between fluoxetine and imipramine groups at both week 4 (F = 3.11, P = 0.04; two-tailed) and week 8 (F = 0.07, P <0.001; two-tailed).

### All samples

Paired sample t-test showed that in the fluoxetine group, TC level had a trend toward the significant decrease from 165.71 ± 35.80 mg/dL (baseline) to 156.71 ± 35.53 mg/dL at week 4 (P = 0.07), and a non-significant decrease to 143.94 ± 44.71 mg/dL at week 8 (P = 0.16). On the other hand, in the imipramine group, TC level had a trend toward the significant increase from 169.10 ± 36.20 mg/dL (baseline) to 178.69 ± 42.78 mg/dL at week 4 (P = 0.07), and a significant increase to 208.69 ± 43.33 mg/dL at week 8 (P < 0.001).

### Male sample

Paired sample t-test showed that in the fluoxetine group, TC level had a non-significant decrease from 154.60 ± 29.39 mg/dL (baseline) to 147.80 ± 27.93 mg/dL at week 4 (P = 0.39), and a significant decrease to 128.70 ± 34.96 mg/dL at week 8 (P = 0.006). On the other hand, in the imipramine group, TC level had a non-significant increase from 159.92 ± 33.18 mg/dL (baseline) to 171.46 ± 39.49 mg/dL at week 4 (P = 0.21), and a significant increase to 194.38 ± 40.42 mg/dL at week 8 (P < 0.001).

### Female samples

Paired sample t-test showed that in the fluoxetine group, TC level had a significant decrease from 181.57 ± 40.29 mg/dL (baseline) to 169.43 ± 43.30 mg/dL at week 4 (P = 0.036), and a non-significant decrease to 165.71 ± 50.58 mg/dL at week 8 (P = 0.86). On the other hand, in the imipramine group, TC level had a non-significant increase from 176.56 ± 37.85 mg/dL (baseline) to 184.56 ± 45.68 mg/dL at week 4 (P = 0.22), and a significant increase to 220.31 ± 43.31 mg/dL at week 8 (P = 0.003).

It should be mentioned that the changes in TC levels were not dependent on age when analyzing the data for all subjects.

### TG levels analysis

Mean TG levels were not significantly different between fluoxetine and imipramine groups at week 4 (F = 1.88, P = 0.20; two-tailed) but were significant at week 8 (F = 1.33, P = 0.002; two-tailed).

### All samples

Paired sample t-test showed that in the fluoxetine group, TG level had a significant decrease from 129.35 ± 26.61 mg/dL (baseline) to 115.88 ± 26.87 mg/dL at week 4 (P < 0.001), and a non-significant decrease to 110.41 ± 48.30 mg/dL at week 8 (P = 0.56). On the other hand, in the imipramine group, TG level had significant increases from 111.73 ± 36.52 mg/dL (baseline) to 128.83 ± 35.27 mg/dL at week 4 (P = 0.005), and to 160.90 ± 56.04 mg/dL at week 8 (P < 0.001).

### Male samples

Paired sample t-test showed that in the fluoxetine group, TG level had a significant decrease from 130.45 ± 32.92 mg/dL (baseline) to 116.18 ± 33.20 mg/dL at week 4 (P = 0.016), and a non-significant decrease to 106.82 ± 50.84 mg/dL at week 8 (P = 0.30). On the other hand, in the imipramine group, TG level had significant increases from 115.42 ± 37.55 mg/dL (baseline) to 127.17 ± 40.54 mg/dL at week 4 (P = 0.013), and to 153.92 ± 42.94 mg/dL at week 8 (P < 0.001).

### Female samples

Paired sample t-test showed that in the fluoxetine group, TG level had a significant decrease from 127.33 ± 9.52 mg/dL (baseline) to 115.33 ± 10.25 mg/dL at week 4 (P = 0.001), and a non-significant decrease to 117.00 ± 46.98 mg/dL at week 8 (P = 0.94). On the other hand, in the imipramine group, TG level had significant increases from 109.28 ± 36.69 mg/dL (baseline) to 129.94 ± 32.50 mg/dL at week 4 (P = 0.036), and to 165.56 ± 64.08 mg/dL at week 8 (P = 0.007). The changes in TG levels were not dependent on age when analyzing the data for all samples.

### Body weight analysis

Mean BW were not significantly different between fluoxetine and imipramine groups at week 4 (F = 0.05, P = 0.41; two-tailed) and week 8 (F = 0.02, P = 0.87; two-tailed).

### All samples

Paired sample t-test showed that in the fluoxetine group, BW was non-significantly decreased from 75.69 ± 7.97 Kg (baseline) to 75.67 ± 8.01 Kg at week 4 (P = 0.88), and to 75.22 ± 8.67 Kg at week 8 (P = 0.20). On the other hand, in the imipramine group, BW had significant increases from 72.53 ± 8.55 Kg (baseline) to 73.95 ± 8.61 mg/dL at week 4 (P < 0.001), and to 75.13 ± 8.34 mg/dL at week 8 (P < 0.001).

### Male samples

Paired sample t-test showed that in the fluoxetine group, BW had non-significant increases from 78.68 ± 7.71 Kg (baseline) to 78.86 ± 7.53 Kg at week 4 (P = 0.44), and to 78.91 ± 7.62 Kg at week 8 (P = 0.85). On the other hand, in the imipramine group, BW had significant increases from 76.85 ± 8.08 Kg (baseline) to 77.92 ± 7.88 Kg at week 4 (P = 0.009), and to 78.92 ± 7.61 Kg at week 8 (P = 0.007).

### Female samples

Paired sample t-test showed that in the fluoxetine group, BW had non-significant decreases from 71.00 ± 6.24 Kg (baseline) to 70.64 ± 6.24 Kg at week 4 (P = 0.25), and to 69.43 ± 7.21 Kg at week 8 (P = 0.14). On the other hand, in the imipramine group, BW had significant increases from 69.58 ± 7.73 Kg (baseline) to 71.24 ± 8.20 mg/dL at week 4 (P < 0.001), and to 72.53 ± 7.97 mg/dL at week 8 (P = 0.008).

It should be mentioned that the changes in BW measures were not dependent on age when analyzing the data for all patients. Using regression analysis did not detect any association between BW (independent variable) and either TC or TG levels (dependent variables) in weeks 0, 4, and 8; no significant association was found between these parameters.

## Discussion

Even though there are some literature on the effects of antidepressants on lipid factors and BW, our study was designed to compare the effects of fluoxetine with imipramine on these factors simultaneously.

In a letters to the editor, Roessner and colleagues reported a case in which TC level was increased in a woman with severe recurrent depression who was taking doxepin [14]. In another study in 1994 by Pollack et al., consumption of nortriptyline after 7 months showed significant elevation in TG and very-low-density lipoproteins (VLDL) levels in 26 elderly patients suffering depression [15].

A case of a hypercholesterolemic female, with post-partum depression, who started receiving fluoxetine two weeks after her labour, was previously reported. The woman’s TC and TG levels was measured prior to as well as 4 and 8 weeks after the initiation of the fluoxetine. Serum TC level was decreased from 242 mg/dL at the baseline to 224 mg/dl after 4 weeks and to 202 mg/dl after 8 weeks [16]. Similarly, triglyceride level was decreased from 516 mg/dL at week 0 to 448 mg/dL at week 4 and further to 404 mg/dL at week 8 [16]. Even though this patient was started on fluoxetine after labour, it should be noted that a part of the change in TC and TG levels may have been due to the nature of pregnancy and the related physiological changes during and after this time.

In a study by Raeder et al. on patients on SSRIs who were based on a cross-sectional survey in Hordaland county in Norway (The Hordaland Health Study ‘97-’99), it was noted that use of most SSRIs may be associated with some metabolic changes. This study reported that sertraline, fluoxetine, or fluvoxamine, in 131 patients, was associated with both abdominal obesity and hypercholesterolemia. However, in 187 patients on paroxetine, the use of the drug showed association with obesity but not with hypercholesterolemia. Additionally, it was noted that there was no association between the use of citalopram in 142 patients with any metabolic factor or lipid profile [12].

On the other hand, association between TCAs (e.g. imipramine) and weight gain has been reported in the literature. Fernstrom et al. examined weight gain in patients taking imipramine 200–250 mg per day for 16 weeks and noted statistically significant increases in weight and body mass index of the patients [17].

Frank et al. reported weight changes in patients with recurrent major depression who were receiving about 215 mg of imipramine per day for over 5 months [18]. This study noted non-significant, minimal changes in body weight during the first 9 weeks of the treatment. On the other hand, different results regarding weight gain have been reported with different agents in SSRI family. Several studies have reported weight loss with short-term use of fluoxetine [19,20]. The literature was reviewed regarding the relative risk for antidepressant-induced weight gain by Fava [21]. This study suggested that TCAs may cause more weight gain than SSRIs. It also noted that long-term use of paroxetine increases weight more than the use of other SSRIs.

Interestingly, a study by Sep-Kowalikowa and colleagues, in 1992, on 329 depressed and neurotic patients noted increases in weight of patients receiving amitriptyline or doxepin but reduction in this factor in those receiving imipramine [22].

Fava et al. evaluated weight changes in patients on 26 to 32 weeks treatment with, paroxetine, sertraline, or fluoxetine. This long-term study noted that paroxetine resulted in significant weight increase, however, sertraline increased and fluoxetine decreased BW in non-significant manners [23].

To our knowledge, no previous study compared the alterations in TC and TG levels as well as BW in patients on imipramine with those on fluoxetine at the same time. The present study compared the effects of short-term use of fluoxetine and imipramine on the above factors in patients with major depressive disorder.

Our study noted a trend toward a significant decrease in TC serum levels of patients on fluoxetine after 4 weeks and a non-significant decrease at the eighth week. Imipramine showed a trend toward a significant increase in TC levels after 4 weeks and a significant increase in this parameter at the eighth week. In addition, our study noted a significant decrease in TG serum levels of patients on fluoxetine after 4 weeks and a non-significant decrease at the eighth week. On the other hand, Imipramine showed significant increases in TG levels at weeks 4 and 8. The changes in TC and TG levels in both groups were in the normal range and did not seem to be clinically significant. However, these changes can become clinically important in patients with high or low normal TC and TG levels or in those with risk factors for developing hypercholesterolemia. There are a few studies that have evaluated the effects of SSRIs or TCAs on lipid profile. TCAs have been noted to have unfavourable effects on TG and low-density lipoprotein cholesterol, probably, because of the weight gain associated with TCAs [24]. Raeder et al. reported that SSRIs as a group were associated with hypercholesrterolemia [12]. However, our study showed the opposite effects for fluoxetine.

It should also be noted that the duration of this study may not have been long enough to show the chronic effects of the studied antidepressants on lipid profile.

There were some other limitations in our study that could have affected the results. Originally, we planned to perform a randomized, double-blind trial. Due to the fact that imipramine was available in tablet form and fluoxetine in capsule form, we were not able to get these medications in the same form at the time. However, since our study primarily was looking into the effects of the drugs on serum TC and TG levels that are not considered as subjective values, we did not think an open-label study would significantly interfere with the results. Another reason was that when we initiated the study, many psychiatrists in Iran preferred TCAs over SSRIs for the treatment of major depression. For this reason, the number of patients on imipramine was much more than those on fluoxetine.

## Conclusions

Clinicians should consider medical conditions of patients and the changes in weight, TC and TG levels of patients, especially those with cardiovascular risk, and any drug-drug interaction before initiating fluoxetine or imipramine. Fluoxetine may be suggested to be used in depressed patients with high normal TC or in individuals with a high risk to develop hypercholesterolemia. Further studies are needed to evaluate the effects of long-term use of imipramine and fluoxetine on lipid profile and BW in depressed patients.

## Competing interest

The authors declare that they have no competing interests.

## Authors’ contributions

ESA was responsible for recruiting patients, visiting and evaluating them; he was also involved in designing the study, analyzing the data and writing the manuscript. PG (the corresponding author) was involved in all aspects of the study from designing the trial and coordination of the study through the statistical analysis and drafting the manuscript. This study was a continuation of MZK’s thesis toward graduation from the residency program in psychiatry (proposal registration #245--78/04/16 at Tehran University of Medical Sciences, Faculty of Medicine); he continued visiting and evaluating patients and was involved in original drafting of the manuscript. MS was consulted for different aspects of the study; he also introduced depressed patients and evaluated them during the study. All authors read and approved the final manuscript.
